# Ant social foraging strategies along a Neotropical gradient of urbanization

**DOI:** 10.1038/s41598-021-85538-2

**Published:** 2021-03-17

**Authors:** Wesley Dáttilo, Ian MacGregor-Fors

**Affiliations:** 1 Red de Ecoetología, Instituto de Ecología A.C., Xalapa, Veracruz Mexico; 2 Red de Ambiente y Sustentabilidad, Instituto de Ecología A.C., Xalapa, Veracruz Mexico; 3 Present Address: Ecosystems and Environment Research Programme, Faculty of Biological and Environmental Sciences, University of Helsinki, Lahti, Finland

**Keywords:** Biodiversity, Community ecology, Urban ecology, Ecology

## Abstract

During the last decades, urbanization has been highlighted as one of the main causes of biodiversity loss worldwide. Among organisms commonly associated with urban environments, ants occupy urbanized green areas and can live both inside and around human settlements. However, despite the increasing number of studies on the ecological dynamics of ant species developed mainly in temperate urban ecosystems, there is still little knowledge about the behavioral strategies that allow ant species to live and even thrive within cities. In this study, we evaluated the role of urbanization in shaping ant communities, including their social foraging, considering built cover as a gradually changing variable that describes an urban gradient. Specifically, we assessed whether species richness, composition, and the proportion of exotic ant species are related to an urban gradient in a medium-sized Neotropical city immersed in a cloud forest context in Mexico. Moreover, we evaluated the social foraging strategies that could promote ant species coexistence in an urban environment. In general, and contrary to our hypothesis, we found no evidence that the built cover gradient affected the richness, composition, or proportion of exotic ant species foraging on food resources, indicating a filtering and simplification of ant communities given by urbanization. Moreover, we show for the first time that urban ant species exhibited a “discovery-defense strategy”, whereby the ant species with the greatest capacity to discover new food resources were those that showed the greatest ability to monopolize it after 120 min of observation, regardless of the type of resource (i.e., tuna or honey bait). Our findings have a direct impact on the knowledge about how urbanization shapes ant communities and behavior, by showing the foraging strategies of ant species that feed on similar food resources present that allows them to coexist in urban environments.

## Introduction

Several studies have shown that urbanization is one of the main causes of the endangerment of wildlife species, along with biological invasions and habitat loss^1,2^. The rapid conversion of landscapes to urban land uses has increased in recent decades worldwide, mainly due to non-urban-to-urban migration, leading to a greater spatial concentration of the human population and infrastructure in the cities^3^. These novel urban ecosystems are characterized by the isolation of their major greenspaces, which remain embedded in and are directly affected by the urban matrix, and where not all species can survive^4^. Therefore, knowing how and why urbanization affects different hierarchical levels of biodiversity (from genes to ecosystems) could improve our understanding of the dynamics of urban ecosystems and their role in the management and conservation of biodiversity.

Ants are a group of organisms commonly associated with urban environments, where they dwell and thrive everywhere from greenspaces to kitchens and hospitals (see ^5^ and references therein). Among the reasons behind ants being successful in cities include the great variety of food resources that they use, together with their nesting behaviors and high tolerance to heat stress^6,7^. Although some ant species cope well with urbanization ^8^, urban ants have been shown to represent a subset of the regional species pools, which is a consistent pattern across taxa^9,10^. It has been estimated that urbanization can lead to the loss of up to 85% of local ant diversity^11^. Such filtering and simplification of ant communities given by urbanization has been shown to be related to the replacement of specialist by common/generalist species, thus increasing the compositional similarity between sites^8,12^. In fact, most studies show that urbanization negatively affects groups of more specialized ants that are more susceptible to disturbance and favors other groups of more generalist and invasive ant species^8,13^. In addition, it is notable that cities are highly heterogeneous, conforming gradients between highly urbanized sites and their greenspace network (e.g., forest fragments, parks, vacant lots, sidewalks, backyards)^14^. Thus, the effect of urbanization on ants seems to be context dependent and is related to how native species can adapt to these new conditions imposed by the environment, as well as with the interactions with some highly abundant exotic and invasive species^15,16^.

In the understanding of how species respond to urbanization in different ways, important research has been focused on the behavioral strategies that allow them to live and even thrive within cities^17^. For ants, two main resource using strategies have been reported in natural environments for species that rely on similar diets and that allow a greater number of species to coexist on small spatial scales. First is the discovery-dominance trade-off ^18,19^, in which some species have the ability to discover food resources and other species to dominate them. In this case, discoverers would be subordinate and less aggressive ant species, with a rapid ability to discover the food resource, while dominant ant species would be those with a high recruitment rate of workers and who have more ways to defend the resource from other species, promoting food resource monopolization^20^. Second is the discovery-defense strategy, which predicts that the first ant species that recruits many workers on a new food resource has the ability to monopolize and defend this resource from other ant species^21^. Additionally, it is notable that different biotic and abiotic factors (e.g., presence of predators and parasitoids, temperature, humidity, habitat structure, amount of food resource, litter disturbance) can alter the outcome of competition among ants in natural environments (reviewed by Parr and Gibb^22^). Therefore, it would be expected for ant foraging strategies to change between contrasting environments. However, despite the accumulated knowledge about the discovery-dominance-trade-off and discovery-defense-strategies in natural environments, to our knowledge, no study has yet assessed whether these strategies could also be used by ants in urban environments.

In this study, we assessed the role of urbanization in shaping ant communities, including their social foraging, considering built cover as a gradually changing variable that describes an urban gradient in a medium-sized Neotropical city immersed in a cloud forest context. Specifically, we evaluated whether species richness, composition, and the proportion of exotic ant species were related to gradients of urbanization. In general, we expected that urbanization would be negatively related to native ant species richness and composition (i.e., a loss of heterogeneity in species composition among sites) and positively related to the proportion of exotic species. This is because an increase in the intensity of urbanization would decrease the area available for nesting and foraging for more specialized native ants^11^ and should favor the more generalist and invasive ant species^8,13^. Afterward, we tested whether ant species richness and composition (including native and exotic species) change between those that discover and those that dominate food resources. In this sense, we evaluated the discovery-dominance trade-off and the discovery-defense strategy as alternative hypotheses that could describe the social behavior of ants that forage on food resources in urban environments.

## Methods

### Study area and sampling sites

**Figure 1:**
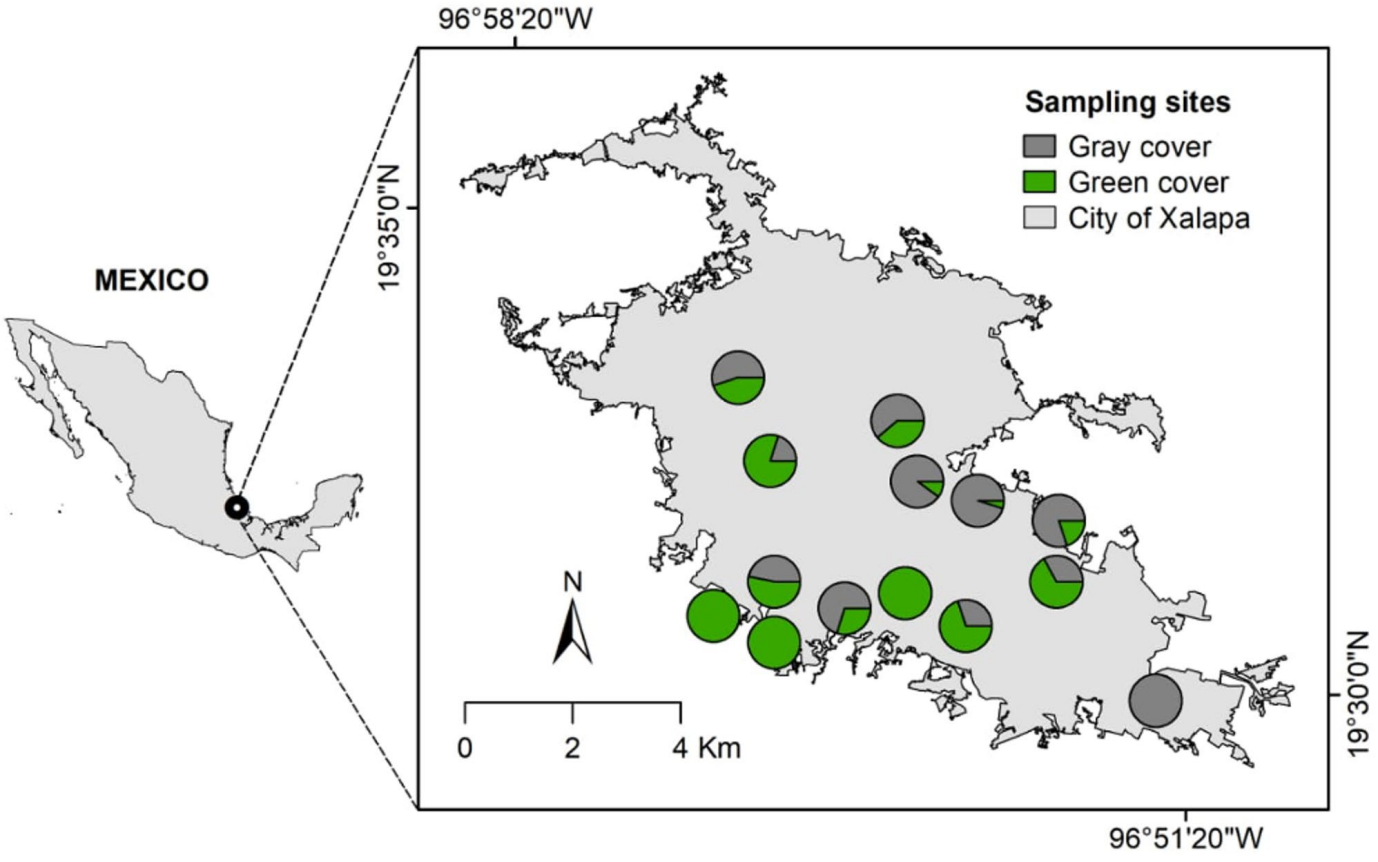
Map of the study site depicting the 14 sampling points that comprise a built cover gradient (from 0 to 100%) in the city of Xalapa, Veracruz, Mexico. The map was drawn in QGIS version 3.16 (https://www.qgis.org) and edited in Inkscape version 0.92 (http://www.inkscape.org).

We conducted this study across an urban density gradient in Xalapa city, Veracruz state capital, Mexico (19°32ʹ37ʺN, 96°54ʹ37ʺW). Xalapa is a small-to-medium-sized city (~ 64 km^2^) with a population of ~ 500,000^23^. The city has an important greenspace network (~ 40% of its territory^24^) that extends across a considerable elevation gradient (1100–1600 m asl). In order to establish an urban density gradient, we placed a grid of 750 × 750 m on top of the polygon of the city and considered the centroid of each quadrant as a potential sampling site, mainly across the city streetscape. Afterward, we relocated those sites that fell on private land to the nearest accessible public site. From the resulting sites, we left out those with security issues. Due to short-distance foraging by ants, we calculated the extent of built cover on circular plots with a 50 m radius at each sampling site using a recently published vegetation cover classification for Xalapa^24^. Briefly, multispectral and panchromatic SPOT satellite images (“AIRBUS TM” SIAP) were geo-referenced, corrected radiometrically, projected to the UTM Zone 14 (Datum WGS 84), and finally merged. The resulting image was classified using Object-Based Image Analysis. The processing consisted of a supervised classification via image segmentation and use of all spatial, textural, spectral, and color space attributes of the image, as well as a Normalized Difference Vegetation Index. The image was classified into three main covers: non-vegetated, herbaceous plants, and woody vegetation (based on > 20 training samples per cover). Validation was pursued through the contrast of 100 points on suitable and available high-quality imagery, with classification overall accuracy of 94% (Kappa coefficient = 0.895) according to a confusion matrix. Finally, the herbaceous and woody vegetation covers were merged into one class, which we considered as variable to establish the gradient for this study. Then, we carried out a map conversion to a raster format in the QGIS program version 3.16 (https://www.qgis.org). Specifically, we selected 12 sampling sites that varied gradual and decreasingly in their vegetation cover to represent a gradient of urbanization density (indicated by the proportion of land deprived of vegetation) of Xalapa. We added two sampling sites located in a peri-urban forest with 100% vegetation cover (Fig. 1).

### Ant sampling

We collected ants in the 14 sampling sites that represent a gradient of urbanization density of Xalapa in November 2019, between 11:00 am and 4:00 pm. At each site, we placed 10 bait stations that consisted of 20-cm-diameter circular plastic plates with two types of bait: honey and tuna. We set honey in five of the stations per sampling site and tuna in the remaining five arranged interspersed in two rows with a separation of at least 10 m between stations. We gathered all ants attracted by both baits 15 min after placing stations and again 2 h after placing them. We used brushes to remove individuals to vials filled with 70% ethyl alcohol. The collected ants were transported to the laboratory at the Instituto de Ecología, A.C. for sorting, mounting, and identification to the lowest possible taxonomic level.

### Data analysis

We performed all analyses below for each type of bait (tuna, honey, and both baits together representing the total community) given that bait type could affect ant behavior. For example, (1) ants foraging on high-carbohydrate diets tend to be more aggressive and exhibit frenzied behavior compared with ants foraging only on nitrogen-rich resources^25–27^ and (2) carbohydrate-rich resources may be used as fuel to increase activity levels, allowing the colony to cover extreme foraging distances^28,29^.

Initially, we evaluated the relationship between the built cover gradient (as an explanatory variable) and ant species richness and the proportion of exotic ant species sampled on tuna and honey bait (as response variables) using generalized linear models (GLMs). Native and exotic species were classified following Dáttilo et al.^30^ for Mexico. Due to the nature of the response variables, a Poisson error distribution was employed for species richness and a quasibinomial error distribution with a logit link function for the proportion of exotic ant species because the data exhibited overdispersion (residual deviance > residual degrees of freedom). Additionally, we used paired t-tests (paired by sampling point) to evaluate if there were differences in the richness of species collected on tuna and honey baits after 15 and 120 min of observation. We then tested for differences in the composition of ants foraging on baits after 15 and 120 min of observation using a permutation multivariate analysis of variance (PERMANOVA) (n = 999 permutations). We also used PERMANOVA to test whether ant composition differed between different categories of built cover. Specifically, we categorized the gradient of urbanization density into three classes (as suggested by MacGregor-Fors^31^): (1) 0 to 33% = low urbanization, (2) 34% to 66% = moderate urbanization, and (iii) 67% to 100% = high urbanization. We performed PERMANOVAs with raw data using the *vegan* package (function *adonis*)^32^.

We used the discovery and dominance ability indices to evaluate the mechanisms that could explain the foraging of urban ants that rely on similar food resources (according to Camarota et al.^21^). For this, we defined discovery ants using the Discovery Ability Index (DAI) by ranking the number of times that an ant species was the first to find the bait (after 15 min of observation). We also ranked the species in terms of the number of times the ant species dominated the baits according to the Dominance Ability Index (DI). In this case, we defined dominant species as follows: when > 10 individuals (workers or soldiers) of the same species were using the resource without the presence of other species after 120 min of observation^33^. We normalized the DAI and DI indices by dividing the values of each species by the highest observed value. Thus, both indices range from 0 to 1, in which the more discovering and dominant ant species had a greater rank value for each index. Note that, the DAI and DI indices are commonly used in the literature, cover important mechanisms explaining ants’ foraging with complementary biological significance and, therefore, allow a direct comparison with the previous studies. We used Spearman’s rank correlation between the DAI and DI indices to test the discovery- dominance trade-off and discovery-defense hypotheses. In this case, a positive correlation between DAI and DI supports the discovery-defense hypothesis, while a negative correlation would agree with the discovery-dominance hypothesis. It is worth noting that correlation tests were performed considering the pool of total species recorded in the city and not for each one of the urbanization gradient sites due to the low species richness that co-occur in the same place and that present values of both DAI and DI indices. All analyses were performed using R^34^.

## Results

We collected a total of 5212 ants belonging to 27 species and four subfamilies (Table 1). Myrmicinae was the most representative subfamily with 16 species recorded (59%), followed by Formicinae (n = 6 species, 22%), Dolichoderinae (n = 4 species, 15%), and Dorylinae (n = 1 species, 4%). *Solenopsis geminata* (Fabricius, 1804) (Myrmicinae) was the only ant species that occurred in all 14 points and represented almost half (43%) of all individuals collected (n = 2255), while *Labidus coecus* (Latreille, 1802) (Dorylinae) was the rarest species, with only one worker collected. Of all the species collected, 18.51% (n = 5) of them were classified as exotic*: Cardiocondyla minutior* Forel, 1899, *Monomorium pharaonis* (Linnaeus, 1758), *Paratrechina longicornis* (Latreille, 1802), *Tapinoma melanocephalum* (Fabricius, 1793), and *Tetramorium simillimum* (Smith, 1851). When we consider the number of individuals collected, we found that 20.77% of them represented exotic ants (n = 1145 ants).

**Table 1:** Number of individuals of native (N) and exotic (E) ant species collected along a built cover gradient in the city of Xalapa, Veracruz, Mexico. Native and exotic species were classified following Dáttilo et al.^30^.

Ant species	Subfamily	Origin	Percentage of built cover													
			0	0	0	20	30	33	47	55	61	70	80	90	95	100
*Atta mexicana* (Smith, 1858)	Myrmicinae	N	–	–	–	–	3	–	–	–	–	–	–	–	–	–
*Brachymyrmex* obscurior Forel, 1893	Formicinae	N	–	1	–	85	4	–	–	1	1	13	19	–	88	–
*Camponotus planatus* Roger, 1863	Formicinae	N	–	–	–	–	1	5	1	–	–	–	3	–	–	–
*Cardiocondyla emeryi* Forel, 1881	Myrmicinae	N	–	–	–	–	–	–	–	–	2	–	–	–	–	–
*Cardiocondyla minutior* Forel, 1899	Myrmicinae	E	–	–	–	–	–	–	–	–	–	6	–	–	–	–
*Dorymyrmex bicolor* Wheeler, 1906	Dolichoderinae	N	–	–	–	27	–	–	10	–	7	1	1	–	15	18
*Forelius damiani* Guerrero & Fernández, 2008	Dolichoderinae	N	2	–	–	60	587	–	–	1	4	–	–	3	135	–
*Labidus coecus* (Latreille, 1802)	Dorylinae	N	–	1	–	–	–	–	–	–	–	–	–	–	–	–
*Linepithema dispertitum* (Forel, 1885)	Dolichoderinae	N	–	–	–	–	–	2	–	31	–	–	–	–	–	–
*Monomorium ebeninum* Forel, 1891	Myrmicinae	N	–	–	–	102	43	–	–	–	–	129	60	2	–	–
*Monomorium pharaonis* (Linnaeus, 1758)	Myrmicinae	E	–	–	–	–	–	–	–	219	1	–	–	–	–	1
*Nylanderia acuminata* (Forel, 1911)	Formicinae	N	–	10	–	–	–	2	–	13	7	1	12	11	1	–
*Nylanderia bourbonica* (Forel, 1886	Formicinae	N	–	8	–	–	–	–	–	–	–	–	–	–	–	–
*Nylanderia steinheili* (Forel, 1893)	Formicinae	N	7	1	–	–	–	–	–	–	3	–	1	–	–	–
*Paratrechina longicornis* (Latreille, 1802)	Formicinae	E	–	–	–	–	–	137	500	1	–	–	2	47	90	127
*Pheidole fallax* Mayr, 1870	Myrmicinae	N	–	13	–	–	–	–	–	–	–	–	–	–	–	–
*Pheidole harrisonfordi* Wilson, 2003	Myrmicinae	N	–	5	–	–	–	–	–	–	–	–	2	–	–	–
*Pheidole insipida* Forel, 1899	Myrmicinae	N	6	–	3	22	2	4	–	37	5	–	–	–	2	–
*Pheidole nubicola* Wilson, 2003	Myrmicinae	N	40	–	12	9	–	2	–	–	12	–	–	41	–	–
*Pheidole psilogaster* Wilson, 2003	Myrmicinae	N	–	–	–	–	12	–	–	–	–	–	–	–	–	–
*Pheidole punctatissima* Mayr, 1870	Myrmicinae	N	–	–	–	1	–	5	–	–	–	–	1	5	–	–
*Pheidole tepicana* Pergande, 1896	Myrmicinae	N	–	–	–	–	–	–	–	1	–	–	23	–	–	–
*Solenopsis aff. picea* Emery, 1896	Myrmicinae	N	–	–	–	–	–	–	–	–	–	1	–	–	–	–
*Solenopsis geminata* (Fabricius, 1804)	Myrmicinae	N	604	141	326	234	49	68	85	3	175	109	221	234	5	1
*Tapinoma melanocephalum* (Fabricius, 1793)	Dolichoderinae	E	–	–	–	–	–	–	–	1	–	–	4	–	–	–
*Temnothorax subdivitus* Mayr 1861	Myrmicinae	N	–	–	–	–	–	–	–	–	–	–	1	–	–	–
*Tetramorium simillimum* (Smith, 1851)	Myrmicinae	E	–	–	–	2	–	–	2	–	5	–	–	–	–	–

**Figure 2:**
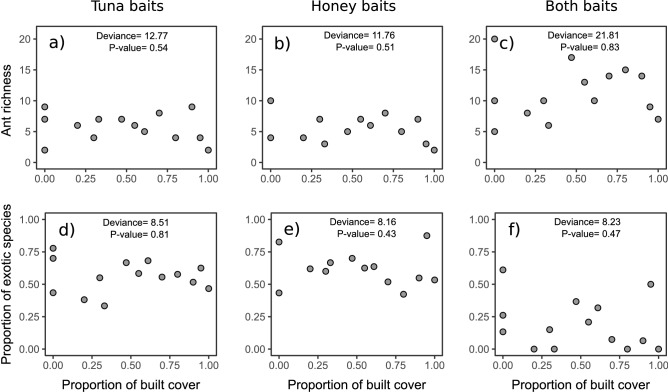
Scatterplots of the relationship between the built cover gradient and ant richness and the proportion of exotic species sampled in tuna, honey, and both baits considered together.

We observed that the built cover gradient was not related to ant species richness collected at tuna baits (GLM deviance = 12.77, *p* = 0.54) or honey baits (GLM deviance = 11.76, *p* = 0.51), as well as when both baits were grouped (GLM deviance = 21.81, *p* = 0.83) (Fig. 2a–c). We also found no evidence that the built cover gradient was related to the proportion of exotic ant species sampled on tuna (GLM deviance = 8.51, *p* = 0.81), honey (GLM deviance = 8.16, *p* = 0.43), or both baits considered together (GLM deviance = 8.23, *p* = 0.47) (Fig. 2d–f). In addition, we found that ant composition did not change across the studied gradient when considering ants collected at tuna (PERMANOVA: F_2,14_ = 0.88, r^2^ = 0.13, *p* = 0.56) and honey baits (PERMANOVA: F_2,14_ = 0.76, r^2^ = 0.12, *p* = 0.72) and for both baits grouped together (PERMANOVA: F_2,14_ = 0.87, r^2^ = 0.13, *p* = 0.61).

For tuna baits, we observed that the total ant richness recorded per sampling site did not change after 15 min (mean ± SD: 3.71 ± 1.48 species) and 120 min (4.21 ± 1.76 species) of observation (paired t-test: t = -0.91, *p* = 0.38). However, for honey baits, we observed that ant richness was lower after 15 min (3.07 ± 1.59 species) when compared with samples of ants collected after 120 min of setting the baits (4.14 ± 1.61species) (paired t-test: t = -2.51, *p* = 0.02). Considering both tuna and honey baits together, we also found no difference in ant richness after 15 (4.85 ± 2.17 species) and 120 min (5.85 ± 1.95 species) of observation (paired *t* test: t = − 1.27, *p* = 0.21).

**Figure 3:**
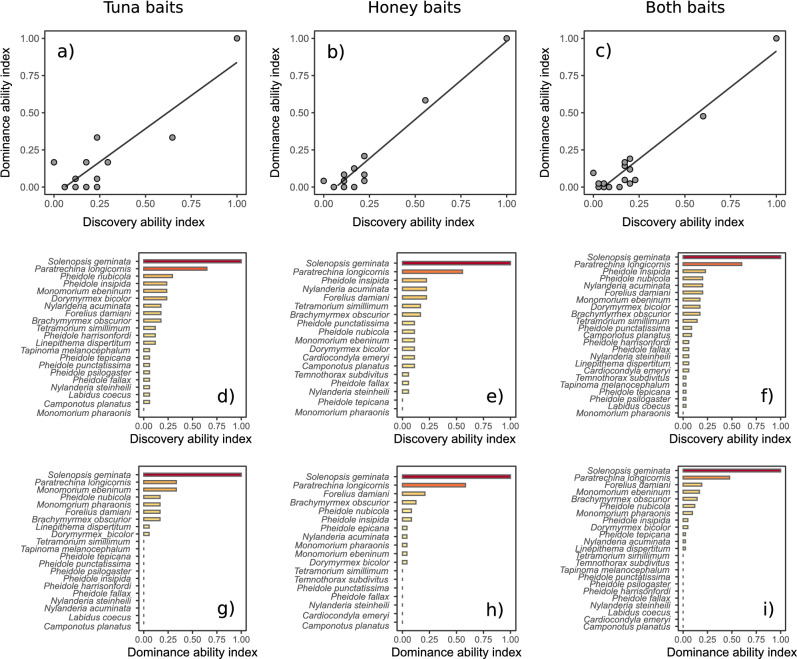
Relationship between the discovery and dominance ability indices involving ants sampled in (**a**) tuna (Spearman’s rank correlation. r^2^ = 0.89, *p* < 0.001) and (**b**) honey baits (Spearman’s rank correlation. r^2^ = 0.88, *p* < 0.001), and (**c**) when both baits were considered together (Spearman’s rank correlation. r^2^ = 0.96, *p* < 0.001). Throughout the letters (**d**)–(**i**) the distribution of the values of the discovery and dominance ability indices for each ant species represented in the relationships of (**a**)–(**c**) are shown.

Finally, we did not record differences in ant species composition after 15 and 120 min of observation, regardless of the type of bait (PERMANOVA: tuna: F_1,28_ = 1.01, r^2^ = 0.03, *p* = 0.43; honey: F_1,28_ = 1.15, r^2^ = 0.048, *p* = 0.35) or when ants sampled using each bait were grouped together (PERMANOVA: F_1,28_ = 1.23, r^2^ = 0.04, *p* = 0.24). In this sense, we observed that few ant species have the ability to discover and dominate baits and that most of them had low values of the discovery and dominance indices (Fig. 3). Of the 20 ant species collected in tuna baits, only 9 (45%) showed the ability to dominate the baits. For honey baits, of the 16 ant species that found the baits, only 10 of them (62.5%) had the ability to dominate baits. The native *S. geminata* (Fabricius, 1804) (Myrmicinae) and exotic *P. longicornis* (Latreille, 1802) (Formicinae) were the only recorded ant species with high values of both discovery and dominance ability indices, regardless of the bait used. Moreover, we found a positive relationship between the discovery ability index and dominance ability index for tuna (Spearman’s rank correlation rho = 0.63, *p* < 0.001), honey (Spearman’s rank correlation rho = 0.62, *p* < 0.001), and both baits considered together (Spearman’s rank correlation rho = 0.65, *p* < 0.001) (Fig. 3a–c).

## Discussion

Contrary to our predictions, we found no evidence that the built cover gradient of the city of Xalapa was related to the species richness, composition, nor the proportion of exotic ant species foraging on tuna and honey baits. Moreover, we observed that, in general, the richness and composition of ants that discover and monopolize different types of food resources did not change between the first 15 and 120 min of observation. Specifically, we show here that ant species with the greatest capacity to discover new food resources are those with highest ability to monopolize it, regardless of the type of resource (tuna and honey baits in the case of this study). These findings show, for the first time, that the discovery-defense hypothesis could be a social foraging strategy for ants in urban environments, rejecting the widely known discovery-dominance hypothesis.

Previous studies have shown that urbanization can pose diverse impacts on ant communities^11,35–37^, including the ecological services they provide (e.g., seed dispersal)^38^. Here, we show that ant species richness was not related to the built cover gradient. This indicates that urbanization acts as a strong environmental filter, only allowing a subset of species from regional pools, but those species that can reach the city appear to be, in general, able to live across the urban landscape^10^. In fact, the ant species found in urban environments are mainly characterized as generalists in terms of their diet and regarding nesting sites^8,39^. Moreover, the ephemerality in the diversity and availability of food resources in urban environments compared with natural ecosystems could lead to few ant species being able to coexist on a small spatial scale^40^, causing the ant richness to remain constant throughout the gradient of built cover studied. Surprisingly, we observed that the proportion of exotic species did not change along the gradient. This suggests that the small subset of both exotic and native species have ecophysiological requirements that allow them to live and thrive in urban environments, since the total richness of ants and the proportion of exotic species remain constant throughout the entire gradient. Interestingly, we also observed that ant composition did not change along the gradient of built cover, regardless of the type of food resource (tuna or honey baits). Despite invasive or exotic ant species often being related to increasing similarity of species composition among areas^41^, here we show that urbanization may be one of the factors that promotes the filtering and simplification of native ant fauna present in cities. Therefore, it seems that both native and exotic ant species have similar life strategies that allow them to dwell under the environmental conditions and with the availability of resources imposed by urbanization.

Additionally, if ants have similar life strategies and coexist in the same environment, they are expected to compete strongly for available resources. Actually, competitive interactions are an important mechanism shaping natural ant communities^6^, and we know that the temporal partitioning of food resources is an important factor that promotes ant species coexistence in natural environments^42,43^. In this study, we observed that the richness of species that visit tuna baits or both baits together did not change between the first 15 and 120 min of observation, indicating that the same number of ant species can discover and further monopolize the new available food resources. However, we found that ant species richness foraging on honey baits was lower after 15 min when compared with after 120 min of observation. Despite the statistical difference in ant species richness, the average number of ants foraging on honey baits was very low in the two time periods (n = 3.07 species in the first 15 min of observation and n = 4.14 species after 120 min of observation). Therefore, despite the high ant richness (n = 27 species) we recorded in the city (considering that Xalapa is at an elevation of ~ 1400 m asl and is immersed in a cloud forest), our results indicate that few ant species can coexist on a small spatial scale and foraging in the same food resources in an urban context.

In relation to ant species composition over time, we did not record differences after 15 and 120 min of observation, regardless of the type of bait used. This indicates that there is a low turnover of species that discover and monopolize baits in urban environments and therefore does not support the discovery-dominance hypothesis (proposed by Fellers^18^). In fact, the discovery-dominance hypothesis appears to be an exception in the literature as shown by Parr and Gibb^19^. Here, we observed the opposite to the discovery-dominance hypothesis (also reported by Santini et al.^44^); rather, we found evidence that adds to the “discovery-defense strategy” occurring in urban systems^21^. Specifically, we found that the first ant species to feed at the baits has the capacity to monopolize it, regardless of the type of food resource. These findings indicate that the discovery phase is an important moment that structures the competition among ants that use similar food resources^21^, since the ant species that firstly arrive at a new resource define whether it will monopolize it. The discovery-defense strategy was already shown to be an important structural mechanism among arboreal ants in the Neotropical savanna^21^, and now we provide further evidence that it can also represent an important mechanism shaping ant foraging in urban environments.

It is notable that in an urban context, where ant species richness tends to be lower compared with natural environments^36,45^, the discovery-defense strategy could be a mechanism of coexistence between ant species because most species have the ability to discover the food resource, but only a few species have the ability to monopolize the resource (Fig. 3). Thus, these findings indicate that many ant species can swiftly find the food resources, but once some ant species with high dominance ability finds the resource, only they will monopolize it. Moreover, we found that only *Solenopsis geminata* (Fabricius, 1804) (Myrmicinae) and *Paratrechina longicornis* (Latreille, 1802) (Formicinae) exhibited high values of both discovery and dominance ability indices regardless of the bait used. Although *S. geminata* is native to Mexico and the exotic species *P. longicornis* is native to Southeast Asia and Melanesia^46^, both species can have extremely large colonies with up to thousands of workers ^46,47^. Moreover, workers of both ant species are widely known for being generalist scavengers^46,47^, which possibly allows them to quickly discover the resource and recruit many workers to defend it from other ant species. Therefore, although many ant species seem to be good resource discoverers across Xalapa, only *S. geminata* and *P. longicornis* showed the ability to be the first to discover food and usually defended it successfully from other ant species, possibly due to their extremely opportunistic foraging behavior (i.e., massive recruitment and aggressive behavior).

In a recent review performed by Santos^5^, the author showed that most of the studies on the biology and ecology of urban ants was carried out in countries of temperate climate and that the amount of theoretical and empirical information available on tropical countries is still limited. Moreover, this author highlights that most studies involving ants in urban environments are focused only on biodiversity loss, arrival of invasive species, and public health (mainly the transmission of pathogens to humans) and often ignore which characteristics allow some species to inhabit urban environments. Here we found that urbanization (measured using a built cover gradient) was not related to shifts in ant species richness or composition, nor with the proportion of exotic/native species, suggesting a filtering and simplification of ant communities present in cities. In addition, this is the first study to show that the discovery-defense strategy could be an important mechanism promoting the coexistence of ant species that inhabit cities, in which the first ant species to discover a new food resource monopolize it successfully over other ant species. Our findings have a direct impact on the existing knowledge of how urbanization shapes ant communities and what strategies the ants that feed on similar food resources present to forage in urban environments. Future studies could examine how the discovery-defense strategy could be extrapolated as a social foraging mechanism involving ants in different cities around the world with different environmental and urbanization contexts.

## Data Availability

Data available on request from the authors.
